# PGK1 Drives Hepatocellular Carcinoma Metastasis by Enhancing Metabolic Process

**DOI:** 10.3390/ijms18081630

**Published:** 2017-07-27

**Authors:** Huijun Xie, Guihui Tong, Yupei Zhang, Shu Liang, Kairui Tang, Qinhe Yang

**Affiliations:** 1 College of Traditional Chinese medicine, Jinan University, Guangzhou 510632, China; huijunxie@jnu.edu.cn (H.X.); zyp6115@jnu.edu.cn (Y.Z.); liangshu2830145@gmail.com (S.L.); tangkarry1991@gmail.com (K.T.); 2 Department of Pathology, Guangdong Provincial Key Laboratory of Molecular Tumor Pathology and School of Basic Medical Sciences, Southern Medical University, Guangzhou 510515, China; tongguihui123@gmail.com

**Keywords:** PGK1, *MYC*, hepatocellular carcinoma, metastasis, Warburg effect

## Abstract

During the proliferation and metastasis, the tumor cells prefer glycolysis (Warburg effect), but its exact mechanism remains largely unknown. In this study, we demonstrated that phosphoglycerate kinase 1 (PGK1) is an important enzyme in the pathway of metabolic glycolysis. We observed a significant overexpression of PGK1 in hepatocellular carcinoma tissues, and a correlation between PGK1 expression and poor survival of hepatocellular carcinoma patients. Also, the depletion of PGK1 dramatically reduced cancer cell proliferation and metastasis, indicating an oncogenic role of PGK1 in liver cancer progression. Further experiments showed that PGK1 played an important role in *MYC*-induced metabolic reprogramming, which led to an enhanced Warburg effect. Our results revealed a new effect of PGK1, which can provide a new treatment strategy for hepatocellular carcinoma, as PGK1 is used to indicate the prognosis of hepatocellular carcinoma (HCC).

## 1. Introduction

Tumor cells mainly obtain energy in a special metabolic condition, which is called the Warburg effect. This is effective in reducing incidence and mortality [1]. The key enzymes in this process can regulate the metabolism, and metabolites produced by tumor cells significantly affect tumor migration and invasion [2]. In solid tumors, possible metabolic exchange is an important dimension of metabolic heterogeneity [3]. However, the metabolic biology of hepatocellular carcinoma (HCC) is yet to be well characterized.

Phosphoglycerate k inase 1 (PGK1) is an important ATP-generating enzyme in the glycolytic pathway [4]. It catalyzes the reversible transfer of a phosphate group from 1,3-bisphosphoglycerate (1,3-BPG) to ADP that produced 3-phosphoglycerate (3-PG) and ATP [4]. PGK1 is very important in tumorigenesis and progression. PGK1 regulates autophagy to promote tumorigenesis [5]. PGK1 is a predictor of poor survival and a novel prognostic biomarker of chemoresistance to paclitaxel treatment in breast cancer [6]. PGK1 appeared to be a predictor of CXCR4 expression, bone marrow metastases, and survival in neuroblastoma [7]. PGK1 is a promoter of metastasis in colon cancer [8], which might be a potential protein biomarker of intracellular oxidative status in human colon carcinoma cells [9]. PGK1 secreted by prostate cancer regulates bone formation at the metastatic site [10]. PGK1 is a potential marker [11] and a promoting enzyme for peritoneal dissemination in gastric cancer [12].

Involved in the glycolytic pathway, PGK1 promotes invasion and metastasis in HCC [13]. Several proteins associates with PGK1 signaling have already been identified, including ornithine transaminase (OTA) [14] and hypoxia inducible factor 1 (HIF-1) [15]. A recent article shows that acetylation at the K323 site of PGK1, as an important regulatory mechanism, promotes its enzymatic activity and cancer cell metabolism [16]. All of these findings indicate that PGK1 is a potential marker for progression in HCC. However, its role in HCC metastasis remains to be further explored.

On the other side, the structure of human PGK1 (hPGK1) has been well understood. hPGK1, as long as 417 amino acids, is a typical hinge-bending enzyme with two similar sized Rossmann fold domains [17]. When the two substrates are bonded, the hinge flexion moves the enzyme into a closed form to allow the substrate to contact [17]. PGK1 inhibits susceptibility to chemotherapeutic drugs in gastric cancer cells and tumor stem cells [18]. PGK1 might be an advisable target molecule for specific immunotherapy of HLA-A2+ colon cancer patients [19]. All these findings indicate the possibility that PGK1 can be a biomarker in the treatment of hepatocellular carcinoma.

*MYC* promotes multiple processes, such as uncontrolled cell proliferation, cell growth, and genomic instability for promoting malignant transformation [20]. Importantly, in order to adapt to the tumor microenvironment, *MYC* also joins the metabolic reprogramming, which is essential for cancer cells [20]. *MYC* as a regulator of PGK1 is essential for proliferation of clear cell renal cell carcinoma cells when its pathway is activated [21]. Proto-oncogene *c-MYC* as an upstream regulator links to the tumor-secreted protein PGK1 in the process of breast cancer development [22]. As for hepatocellular carcinoma, there is a hypothesis that *c-MYC* mainly regulates controlling PGK1 expression [16]. Therefore, the role of *MYC* regulates the metabolic function of PGK1 in HCC which needs to be further studied.

Here we utilized a public database and HCC cell lines to assess the expression and to evaluate the significance of PGK1. The results showed that PGK1 not only promoted HCC cell lines proliferation, but also boosted metastasis via *MYC*-dependent PGK1 expression to modulate the HCC cells metabolism. Moreover, PGK1 was overexpressed in HCC, and it was associated with poor prognosis and worse malignancy. These results recommend a new view of PGK1 in HCC development.

## 2. Results

### 2.1. Phosphoglycerate Kinase 1 (PGK1) Promotes Proliferation in Hepatocellular Carcinoma (HCC) In Vitro

**Figure 1:**
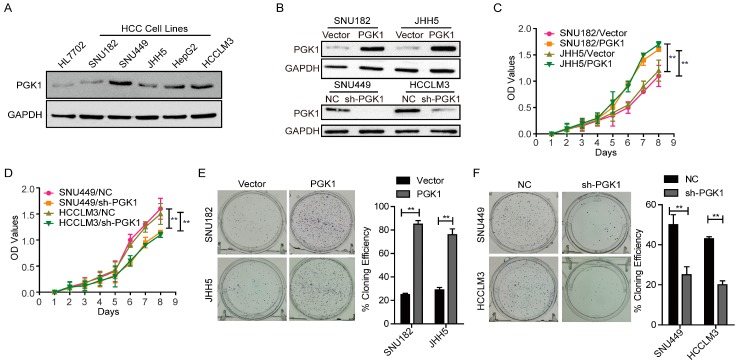
Effect of Phosphoglycerate Kinase 1 (PGK1) on the proliferation of Hepatocellular Carcinoma (HCC) cells. (**A**) Endogenous expression of PGK1 in nomal and five HCC cell lines by Western blotting. Glyceraldehyde 3-phosphate dehydrogenase (GAPDH) was used as internal control. (**B**) Transfection efficiency of PGK1 overexpress and knockdown in HCC. GAPDH was used as internal control. (**C**) Effect of PGK1 on proliferation of SNU182 and JHH5 cells by cell counting kit-8 (CCK8) assay. (**D**) Effect of PGK1 knockdown on proliferation of SNU449 and HCCLM3 cells by CCK8 assay. (**E**) Effect of PGK1 on proliferation of SNU182 and JHH5 cells by plate colony formation assay. (**F**) Effect of PGK1 knockdown on proliferation of SNU449 and HCCLM3 cells by plate colony formation assay. ** *p* < 0.01. NC, negative control; sh-PGK1, short hairpin RNA inhibiting PGK1 gene.

Concerned with the function of PGK1 protein, Western blotting assays were used to detect endogenous expression of PGK1 in a panel of HCC cell lines and a normal liver cell line, HL7702. The result indicated that SNU449 and HCCLM3 expressed higher levels of PGK1 protein compared to HL7702 cells. While among the HCC cell lines examined, SNU182 and JHH5 expressed the lowest, which was similar to HL7702 (Figure 1A). According to the results of endogenous expression of PGK1 in HCC cell lines, SNU182 and JHH5 were selected for the overexpression of PGK1, SNU182/Vector and JHH5/Vector were used as the normal control; SNU449 and HCCLM3 were selected for knocking down PGK1, then we examined the transfection efficiency by Western blotting with SNU449/NC and HCCLM3/NC as normal controls (Figure 1B).

Cell counting kit-8 (CCK8) assay displayed that the forced PGK1 expression significantly increased the ability of SNU182 and JHH5 cells to proliferate (*p* < 0.01, Figure 1C). As PGK1 was knocked down in SNU449 and HCCLM3 cells, the proliferation rate evidently decreased (*p* < 0.01, Figure 1D). These results were validated by the plate colony formation assay (*p* < 0.01, Figure 1E,F). Thus, PGK1 is adequate to promote the proliferation of HCC cells.

### 2.2. PGK1 Is Effective in Promoting Tumor Metastasis In Vivo

**Figure 2:**
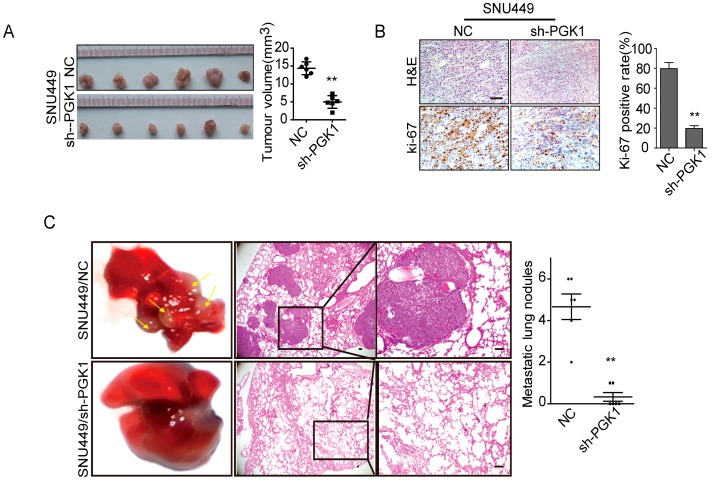
Effect of PGK1 on proliferation and metastasis in vivo. (**A**) Effect of PGK1 knockdown in HCC cell proliferation in vivo. PGK1 knockdowning SNU449 cells and control cells were implanted subcutaneously into nude mice to perform xenograft assay (*n* = 6). Tumor volumes were measured on the indicated days. Data points are presented as the mean tumor volume ± SD. (**B**) Histopathological analysis of xenograft tumors. The tumor sections were stained with H&E or subjected to IHC staining using an antibody against Ki-67. (**C**) Effect of PGK1 knockdown on CRC metastasis in vivo. 1 × 10^6^ cells knockdowning PGK1 or control vector were injected into each nude mouse through tail vein. The number of lung metastasis nodules was counted under the microscope. Error bars represent mean ± SD. The tumor sections were stained with H&E. ** *p* < 0.01.

To investigate the function of PGK1 in tumor growth in vivo, SNU449/sh-PGK1 cells or SNU449/negative control (NC) cells were subcutaneously implanted into nude mice (*n* = 6) and monitored tumor growth. Notably, knocking down PGK1 obviously inhibited tumor growth in vivo (*p* < 0.01, Figure 2A). Additionally, compared to control groups, sh-PGK1 decreased tumor proliferation indices by Ki-67 expression (*p* < 0.01, Figure 2B).

Furthermore, we performed tail vein injecting assay (*n* = 6) in nude mice to evaluate the effect of PGK1 in tumor metastasis in vivo. We injected SNU449/sh-PGK1 cells or SNU449/NC cells into node mice’s tail veins. As shown in Figure 2C, the metastatic lung nodules of sh-PGK1 group was larger than the control group (*p* < 0.01). These results declared that PGK1 is sufficient to promote tumor metastasis in vivo.

### 2.3. MYC-Dependent PGK1 Modulates Metabolic Reprogramming of HCC Cells

**Figure 3:**
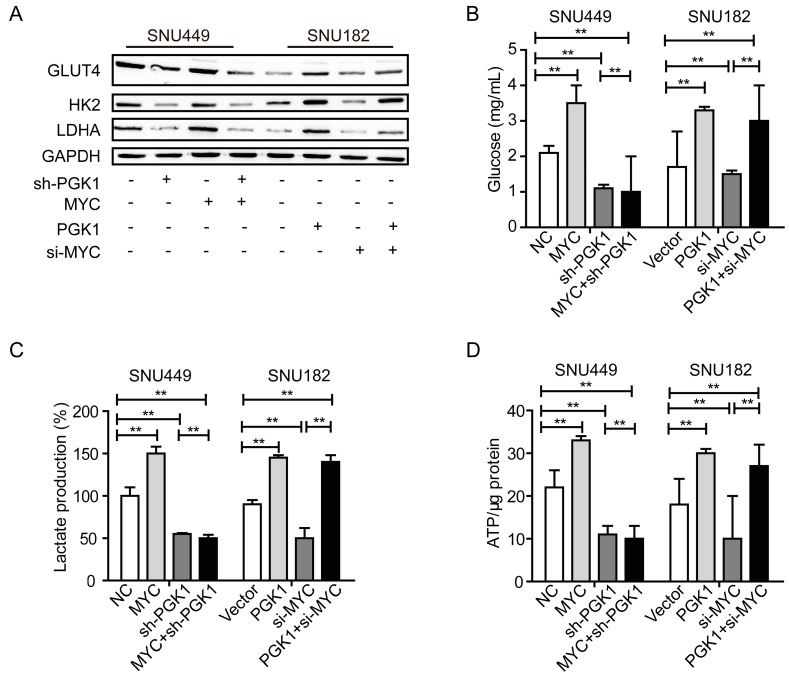
PGK1 modulated Warburg effects of HCC cells. (**A**) Western blot analysis of the expression of GLUT4, hexokinase 2 (HK2), and lactate dehydrogenase A (LDHA of PGK1, sh-PGK1, si-*MYC*, or *MYC* cells. GAPDH was used as internal control. (**B**) Glucose uptake levels of SNU449 and SNU182 cells with PGK1 overpression, sh-PGK1, si-*MYC*, or *MYC* overpression. (**C**) Lactate production of SNU449 and SNU182 cells with PGK1 overpression, sh-PGK1, si-*MYC*, or *MYC* overpression. (**D**) ATP levels of SNU449 and SNU182 cells with PGK1 overpression, sh-PGK1, si-*MYC*, or *MYC* overpression. ** *p* < 0.01.

PGK1 is a major metabolic enzyme in the glycolysis pathway, and *c-MYC* is the main regulator in controlling PGK1 expression [16]. Therefore, we explored the rescue experiment to detect the metabolic process in HCC. Western blotting analysis was performed to assess the expression of solute carrier family 2 facilitated glucose transporter member 4, SLC2A4 gene (GLUT4), hexokinase 2 (HK2), and lactate dehydrogenase A (LDHA) in the rescue experiment. Results revealed that PGK1 significantly increased the expression of GLUT4, HK2, and LDHA in SNU182 cells, while si-*MYC* could not rescue this effect (Figure 3A). Knockdown of PGK1 decreased the expression of GLUT4, HK2 and LDHA in SNU449 cells, while *MYC* could not rescue this effect (Figure 3A). However, knockdown of *MYC* decreased the expression of GLUT4, HK2, and LDHA in SNU182 cells; while PGK1 could rescue this effect (Figure 3A). These results support that the knockdown of *MYC* decreases the expression of GLUT4, HK2, and LDHA by PGK1 inhibition.

Extracellular acidification measurement was used to determine the metabolic condition of HCC. Knockdown of PGK1 decreased cellular glucose uptake in SNU449 cells, while *MYC* could not rescue this effect (*p* < 0.01, Figure 3B). PGK1 significantly increased glucose uptake in SNU182 cells, while si-*MYC* could not rescue this effect (*p* < 0.01, Figure 3B). However, *MYC* significantly increased glucose uptake in SNU449 cells, while sh-PGK1 could rescue this effect (*p* < 0.01, Figure 3B). The knockdown of *MYC* decreased cellular glucose uptake in SNU182 cells, while PGK1 could rescue this effect (*p* < 0.01, Figure 3B). Analogous results were observed in lactate production and ATP levels (Figure 3C,D). *MYC* induces PGK1 to improve metabolic efficiency in HCC. We hypothesize that this improved metabolic efficiency is one of the mechanisms by which PGK1 promotes the proliferation and metastasis of HCC.

### 2.4. PGK1 Is Overexpressed in Human Hepatocellular Carcinoma Specimens

**Figure 4:**
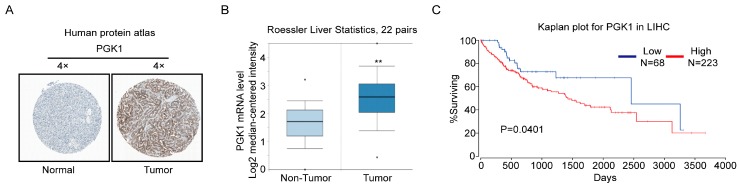
PGK1 is upregulated in human hepatocellular carcinoma specimens. (**A**) PGK1 expression in normal liver tissue and hepatocellular carcinoma specimens. Images were taken from the Human Protein Atlas online database. (**B**) Oncomine data showing PGK1 expression in normal vs. tumor of liver (*n* = 22). (**C**) Kaplan Meier survival analysis of HCC patients with high and lowPGK1 expression based upon data generated by the TCGA Research Network (*p* = 0.0401). ** *p* < 0.01. LIHC, Liver hepatocellular carcinoma.

The clinical relevance of PGK1 expression was determined by three online databases. First, we analyzed the PGK1 protein expression in clinical specimens from the human protein atlas (www.proteinatlas.org). We found that PGK1 had the positive strong expression in HCC, and negative weak expression in normal liver (Figure 4A). Secondly, in the Roesser liver database (Compendia Biosciences, www.oncomine.org), PGK1 mRNA level was superior in HCC tissues to normal liver tissues (*p* < 0.001, *n* = 22, Figure 4B). At last, we exploited the TCGA Research Network: http://cancergenome.nih.gov/ to evaluate the results of survival data. The results showed that patients with high PGK1 expression may have a significantly shorter 10-year survival time (*p* = 0.0401, Figure 4C). These results suggest that PGK1 upregulation in HCC tissue is closely related to the prognosis of HCC patients.

## 3. Discussion

Metabolic reprogramming was thought to be a sign of cancer [23], and had been a popular research field for the past decade. The Warburg effect was not only beneficial to the growth of cancer cells, but also conducive to tumor migration and invasion.

The carcinogenic signaling pathway directly promoted the acquisition of nutrients and promoted the decomposition of carbon into macromolecules (lipids, proteins, and nucleic acids) when nutrient-enriched for the proliferation of cancer cells. These net effects of conducts were expected to strengthen growth and cell proliferation [3]. In the hierarchical structure of the tumor alteration pathway, the mutations of *MYC*, TP53, Ras-related oncogene, LKB1-AMP kinase (AMPK) and PI3K kinase (PI3K) signaling pathway were always invoved in glucose and glutamine metabolism [3]. The increase in *MYC* caused a number of metabolic effects by reprogramming gene expression, including promoted glycolysis, partial activation by LDHA transcription [24], improved mitochondrial biogenesis [25], and increased glutamine catabolism [26,27], eventually achieving biomass assimilation. The intersection of glucose and glutamine in many aspects reflected their richness and they both could enter the central metabolic multiple nodes. Glutamine was necessary for growth, as it provided two nitrogen atoms to synthesize hexosamine, nucleotides, and amino acids [28].

However, for resisting the physical environment of solid tumors, cancer cells must optimize nutritional use due to the scarce resources. Some works highlighted the importance of culturing cells and metabolic flexibility in vivo. For instance, in colon cancer cells, the deprivation of glucose or subcutaneous space in mice grown in a harsh environment caused selective stress on KRAS mutations [29].

Here, KRAS mutation allowed the cells to tolerate low glucose conditions. Cancer cells usually employ a nutrient to pack another nutrient normally provided into the metabolite pool, which can recombine their metabolism to remunerate the loss of glucose or glutamine in the culture [30,31,32]. As a means of sustaining growth, high-throughput screening showed that long-term exposure to low glucose cells required oxidative phosphorylation [33]. Similarly, a subset of lymphomas was preferentially used and it perfered to depend on oxidative metabolism in a classic glycolytic phenotype [34]. Thus, hypoxia-inducible factor (HIF1) activation, lactate released and redox production, these metabolic transitions caused by cancer cells, changed by associating with the development of invasive cancers [2]. A mathematical model provided an acid-mediated hypothesis of invasion of tumor [35]. Through this model, due to the additional glucose production and glucose metabolism changes, the H+ flow concentration gradient generated adjacent to the normal organization. The release of cathepsin B and other proteolytic enzymes to normal cells caused cellular death and extracellular matrix degradation, resulting in chronic exposure to the acidic microenvironment of the surrounding normal tissue. All of these allow cancer cells to invade adjacent normal tissues [2]. In addition, glycolytic enzymes are very important to the migration of tumor cells. GLUT4 is a member of the solute carrier family 2 (promotes glucose transporter) family and transmits glucose through the cell membrane. HK2 is the key enzyme for the first step in most glucose metabolic pathways. As a key metabolic enzyme, lactate dehydrogenase (LDH) catalyzes pyruvate conversely to lactate. LDHA is a subunit of LDH. LDHA was essential for maintaining glycolysis and improving the cancer cells’ invasive activity [2].

**Figure 5:**
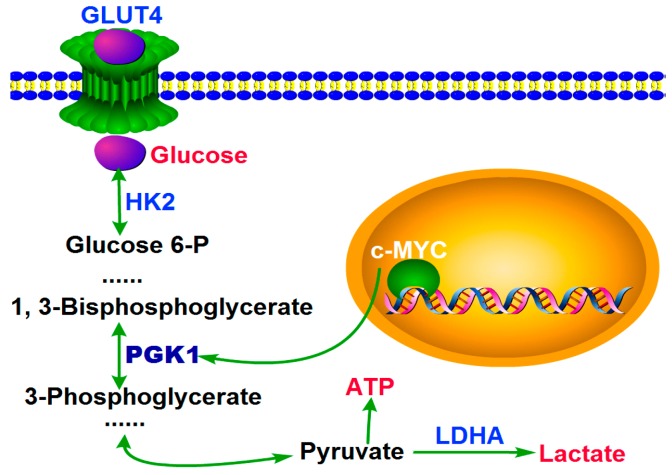
A model for the role of PGK1 in human hepatocellular carcinoma. PGK1 had an important role in metabolic reprogramming induced by *MYC*, leading to an enhanced Warburg effect.

Here we showed that PGK1 was overexpressed in human HCC tissues, and *MYC* induced PGK1 to improve the metabolic efficiency in HCC. We demonstrated that, in HCC cells, more and more glucoses were transported into cells by improved GLUT4, which induced by *MYC*-dependent PGK1. Then with the over expressed of HK2 and LDHA (they are both the key enzymes in glycolysis) induced by *MYC*-dependent PGK1, the rate of glycolysis was accelerated. After that, more and more ATP and lactate were produced. Finally, a large amount of ATP provided energy required for HCC proliferation, and lactate promoted metastasis and had a interrelation with poor hepatic carcinoma overall survival and patient prognosis (Figure 5). Our results demonstrated that oncogenic PGK1 enhanced expression of GLUT4 to stimulate glucose uptaken; on the other hand, PGK1 utilizated the glucose by anabolic pathways. *MYC* upregulated PGK1, resulting in more metabolic product and ultimately cell survival and growth. Moreover, the structure of Human PGK1 was well understood. Our findings strongly suggest that targeting PGK1 is a therapeutic strategy for HCC.

## 4. Materials and Methods

### 4.1. Cell Culture

Human HCC cell lines (SNU182, SNU449, JHH5, HepG2, and HCCLM3) and normal liver cell line HL7702 were acquired from Cell Bank of Type Culture Collection (Shanghai City, China). The cells were cultured in DMEM (Hyclone, Los Angeles, CA, USA) with 10% FBS under 5% CO_2_ at 37 °C.

### 4.2. Cell Transfection with siRNAs and Plasmids

All the primers for PGK1 and *MYC* detection assays were purchased from Ribobio. Transfection of *MYC* siRNA and negative control (NC) were conducted via siRNA kit (RIBOBIO, Guangzhou, China) according to the manufacturer’s protocol.

The steps of PGK1 and *MYC* overexpression are as follows: the CDS full length of human PGK1 and *MYC* genes was synthesized and cloned into Pez-Lv105 vector by GeneCopoeia (Guangzhou, China). The vectors were transfected into lentiviral packaged 293T cell lines. Then 1 mL of viral supernatant (containing 4 Attograms (Ag) of polybrene) was added into HCC cell lines for 14 days.

To eliminate PGK1, a lentivirus with shRNA vector targeting PGK1 was transfected into HEK293T cells, and 1 mL of virus supernatant (containing 4 Ag of polybrene) was added to the HCC cell line. Western blotting detected PGK1 expression after 72 h.

### 4.3. Western Blotting

First, cells were lyzed on ice in a radioimmunoprecipitation (RIPA) buffer with a protease inhibitor then quantified by quinolinic acid (BCA) assay. Second, SDS-PAGE isolated a total of 50 μg of protein lysate, which was then transferred to a PVDF membrane (Millipore Corp, Billerica, MA, USA). Third, the membrane was blocked with 5% fat-free milk, then incubated with PGK1 (ABclonal Biotech Co., Woburn, MA, USA, 1:100), *MYC* (ABclonal Biotech Co., Woburn, MA, USA, 1:100), LDHA (ABclonal Biotech Co., Woburn, MA, USA, 1:100), HK2 (ABclonal Biotech Co., Woburn, MA, USA, 1:100), GLUT4 (ABclonal Biotech Co., Woburn, MA, USA, 1:100), or glyceraldehyde 3-phosphate dehydrogenase (GAPDH) (ABclonal Biotech Co., Woburn, MA, USA, 1:1000) at 4 °C overnight. After that, the membranes were incubated with secondary antibodies. Then, ECL test reagent (Fudebio, China) was used to show the bands.

### 4.4. Cell Proliferation Assay

8 × 10^2^ cells were suspended in 100 μL medium, then seeded in 96-well plates and incubated for seven days. 10 μL of CCK8 (Dojindo Molecular Technologies, Inc., Kumamoto, Japan) added in the new 100 μL medium was added to every well and the cells were incubated with 5% CO_2_ at 37 °C for 2 h. Then the absorbance values were measured with a microplate reader set at 570 nm. Each experiment was conducted for three times.

### 4.5. Plate Colony Formation Assay

2 × 10^2^ cells were suspended in 2 mL medium, then seeded in 6-well plates and incubated at 37 °C with 5% CO_2_ for 14 days, then stained with hematoxylin. After that, counting colonies containing more than 50 cells. Last, a calculated with the formula—plate clone formation efficiency = (number of colonies/number of cells inoculated) × 100% was applied.

### 4.6. Proliferation and Metastasis in Mouse Model

For assessing tumor growth in vivo, 2 × 10^6^ cells were subcutaneously injected into the left abdomen or right abdomen of the six-week-old non-obese diabetic (NOD)/severe combined immunodeficiency (SCID) mice, each group had six mice. After 28 days, the tumor was measured with a caliper to assess the tumor volume.

For assessing tumor transfer in vivo, 2 × 10^6^ cells were injected into the tail vein of six-week-old NOD/SCID mice, each group had six mice. After eight weeks, the animals were euthanized; the thoracic, peritoneum, and peritoneal cavity of the various organs removed, washed, fixed, and then underwent pathological examination. The number of metastatic lung nodules was determined.

### 4.7. Glucose Uptake, Lactate Production, ATP Level Detection

In glucose uptake assay, first, cells were cultured in 6-well plates. Then according to the manufacturer’s protocol, glucose uptake was determined using a glucose assay kit (Biovision Inc., Milpitas, CA, USA).

In lactate production measurement, according to the manufacturer’s protocol, first cells were seeded into 96-well plates with phenol red-free medium, then determined using a lactate assay kit (Biovision Inc., Milpitas, CA, USA).

In ATP levels assay, an ATP assay kit (Biovision Inc., Milpitas, CA, USA) was used according to the manufacturer’s protocol.

### 4.8. Analysis of PGK1 Expression in Human HCC

In HCC tissues and normal tissues, PGK1 protein expression was determined from the human protein atlas (www.proteinatlas.org). HCC PGK1 gene expression was determined through analysis of Roessler and TCGA databases, which are available through Oncomine (Compendia Biosciences, www.oncomine.org) and UCSC (http://xena.ucsc.edu/getting-started/). Survival analysis for the gene expression data were performed using OncoLnc [36].

### 4.9. Statistical Analysis

Statistical analysis was carried out by SPSS 13.0 software. Utilizated Student’s *t*-test to compare the groups in the cell experiments. The data were expressed as the mean ± SEM of the three independent experiments. *p* < 0.05 was considered statistically significant.
